# Phytogenic Feed Additives in Poultry: Achievements, Prospective and Challenges

**DOI:** 10.3390/ani11123471

**Published:** 2021-12-06

**Authors:** Nedra Abdelli, David Solà-Oriol, José Francisco Pérez

**Affiliations:** Animal Nutrition and Welfare Service (SNIBA), Department of Animal and Food Science, Facultat de Veterinària, Universitat Autònoma de Barcelona, 08193 Bellaterra, Spain; David.Sola@uab.cat (D.S.-O.); josefrancisco.perez@uab.cat (J.F.P.)

**Keywords:** phytogenics, performance, digestibility, microbiota, immunity, oxidant status, organic acids, microencapsulation, poultry

## Abstract

**Simple Summary:**

Plant secondary metabolites and essential oils also known as phytogenics are biologically active compounds that have recently attracted increased interest as feed additives in poultry production, due to their ability to promote feed efficiency by enhancing the production of digestive secretions and nutrient absorption, reduce pathogenic load in the gut, exert antioxidant properties and decrease the microbial burden on the animal’s immune status. However, the mechanisms are far from being fully elucidated. Better understanding the interaction of phytogenics with gastrointestinal function and health as well as other feed ingredients/additives is crucial to design potentially cost-effective blends.

**Abstract:**

Phytogenic feed additives have been largely tested in poultry production with the aim to identify their effects on the gastrointestinal function and health, and their implications on the birds’ systemic health and welfare, the production efficiency of flocks, food safety, and environmental impact. These feed additives originating from plants, and consisting of herbs, spices, fruit, and other plant parts, include many different bioactive ingredients. Reviewing published documents about the supplementation of phytogenic feed additives reveals contradictory results regarding their effectiveness in poultry production. This indicates that more effort is still needed to determine the appropriate inclusion levels and fully elucidate their mode of actions. In this frame, this review aimed to sum up the current trends in the use of phytogenic feed additives in poultry with a special focus on their interaction with gut ecosystem, gut function, in vivo oxidative status and immune system as well as other feed additives, especially organic acids.

## 1. Introduction

Poultry production is undergoing a continuous challenge to develop management strategies to optimize chickens’ efficiency while limiting food safety concerns. Traditionally, antimicrobials have been widely used for improving health and growth performance in poultry; however, the increased public awareness about the risk of developing cross-resistance of pathogens to antibiotics has resulted in the gradual removal of antibiotics for therapeutic and prophylactic uses in food animals [1]. The shift away from antibiotic supplementation has resulted in a tremendous growth in research focusing on the implementation of effective alternative control methods, management and dietary amendments aiming to improve animal health, welfare, and productivity. A wide range of feed additives including a broad spectrum of essential oils and related compounds from botanical sources to organic acids [1,2], as well as probiotics and prebiotics [3], chemicals such as aldehydes [4], bacteriophages [5], zinc oxide [6], exogenous enzymes [7] and competitive exclusion products [8] have been used in animal production. Particularly, phytogenic feed additives (PFAs), also popularly referred as phytobiotics or botanicals, have gained an increasing interest as cost-effective feed additives with proven positive effects on broiler chickens’ intestinal health. Indeed, antioxidative, immunomodulatory and growth-promoting effects have also been largely described in the literature. Therefore, the aim of the present review is to summarize the main results of some recent studies evaluating the effect of PFAs supplementation on the major components of bird gastrointestinal health and functionality, with special focus on nutrient digestibility, gut microbiota, immune system, oxidative status and growth performance of broilers and laying hens.

## 2. Gastrointestinal Health and Functionality

The regulation of gastrointestinal tract (GIT) function involves complex interactions among six major components, including the diet, effective digestion and absorption, normal and stable microbiota, effective immune status, gut mucosa, and neuroendocrine and motor function of the gut [9]. Both, diet composition (ingredients, nutrients and additives) and form, including structure and particle size, may affect the GIT functionality, especially through modulating the immune system and intestinal microbiota [9]. In fact, some dietary factors such as certain types of dietary fiber, trypsin inhibitors, phytate, lectins, undigested protein in the distal gastrointestinal tract, mycotoxins, as well as diets with poor nutrient balance, may affect the inflammatory process by modulating both pro-inflammatory and anti-inflammatory mechanisms [10] and thereby, disrupt the structural and functional integrity of the gut [11]. Conversely, feed additives such as phytobiotics, organic acids, enzymes, prebiotics, probiotics [12,13,14], functional foods and nutraceuticals [15] may play key roles in promoting overall health and growth performance.

The main attributes of an effective GIT functionality is an optimal digestion and absorption, maintenance of fluid and electrolyte balance, and elimination of waste products as well as maintenance of a barrier against antigens and pathogens [9]. The gastrointestinal compartments of healthy chickens are densely harbored by complex microbial communities which provide both nutrition and protection for the animal. Commensal microbiota may stimulate the development of immune system including the mucus layer, epithelial monolayer, the intestinal immune cells (e.g., cytotoxic and helper T-cells, immunoglobulin producing cells and phagocytic cells), and the lamina propia; thereby allowing to form a protective barrier between the host and the microbes [16]. Moreover, the microbiota of the distal gut (i.e., caeca and colon) uses the undigested feed to produce vitamins (e.g., vitamin K and vitamin B groups), amino acids, and short chain fatty acids (SCFAs: acetic acid, butyric acid and propionic acid) which eventually become available for the host. These SCFAs are considered of great interest for the host, for their bacteriostatic properties allowing to eliminate foodborne pathogens, such as *Salmonella* spp. [17], and as a source of energy which can stimulate gut epithelial cell proliferation and the gastrointestinal absorption surface [18].

On the other hand, impaired digestion and absorption results in a delivery of excess nutrients (such as starch, protein and fat) to the distal segments of the gastrointestinal tract, which induces alterations in the GIT microbial community resulting in qualitative and/or quantitative imbalance of normal microbiota in the small intestine. This imbalance is characterized by proliferation of pathogens which may lead to a sequential reaction in the GIT, including reduced intestinal barrier function (e.g., thinning of intestinal wall) and poor nutrient digestibility; and therefore, increasing the risk of bacterial translocation and inflammatory responses [19]. These negative effects on the symbiotic interactions between host and microbe lead to adverse effects on feed efficiency, productivity, and health of chickens [16].

The maintenance of GIT integrity is also crucial to ensure effective immune system as GIT is considered the largest organ of the immune system [20], that plays pivotal physiological role as barrier against antigens and pathogens. On the other hand, the GIT also possesses a neuroendocrine function through conveying neuroendocrine signals to the brain during digestion aiming to align the digestive and absorptive capacity of the GIT with the amount and composition of ingested food [11]. Therefore, it secretes gastrointestinal peptide hormones, such as gastrin, secretin and cholecystokinin in response to nutrient, neural or hormonal stimulation as well as by metabolic products of the gut microbiota. These peptide hormones are involved in the digestive and absorptive function of the GIT such as the regulation of gastric acid and pancreatic secretion, release of bile from the gall bladder and gut motor activity [9].

The diet is a main factor modulating the composition and the metabolic activity of the GIT microbiota [21]. In this regard, several feed additives have been developed focusing on enhancing immune response, reducing pathogen load in the GIT, promoting the colonization of the GIT with beneficial bacteria and stimulating digestion and absorption. The current review will discuss the effects of PFAs on the different components of GIT functionality.

## 3. Phytogenics as an Alternative to Antimicrobials in Poultry Feeding

A broad range of plants derived products may fall under the category of phytogenic feed additives. They may be classified either based on their origin (the part of the plant) into herbs (products from flowering, non-woody, and non-persistent plants from which leaves and flowers are used) and spices (non-leaf parts of plants, including seeds, fruits, bark or root with intensive taste or smell); or depending on the process used to derive the active ingredients as essential oils (EOs: volatile lipophilic substances obtained by cold extraction or by steam or alcohol distillation) and oleoresins (extracts derived by non-aqueous solvents). The bioactive components of PFAs are secondary metabolites being polyphenols the main group. Other bioactive compounds include terpenoids (monoterpenes, steroids…), phenolics (tannins), glycosides, and alkaloids [21]. The composition and concentration of these bioactive substances may vary according to several factors including the plant, parts of the plant, geographical origin, harvesting season, climatic conditions, processing techniques such as extraction, distillation and stabilization as well as storage conditions [22,23].

In recent years, PFAs have attracted an increasing attention as natural alternative to antibiotic growth promoters (AGPs) in poultry production which can be included in feeds as dried, solid, and ground form, or as extracts (crude, concentrated and purified) [24]. A wide variety of herbs and spices (thyme, oregano, cinnamon, rosemary, marjoram, yarrow, garlic, ginger, green tea, black cumin, coriander, among others) as well as EOs (from thymol, carvacrol, cinnamaldehyde, garlic, anise, rosemary, citruses, clove, ginger) have been used in poultry, individually or mixed, for their potential application as AGP alternatives [24]. Although the repertoire mechanisms of action of PFAs is not fully elucidated in poultry, one of their primary mode of action is related to their antimicrobial effects which allow controlling potential pathogens [25]. The results obtained in some recent studies will be reported with more details in the current review. However, some authors reported no positive effects of PFAs inclusion [26]. This discrepancy may be attributed to several factors, including the inherent variability of the botanic composition, as well the variability of the animal scenarios, environmental, management and sanitary conditions (i.e., including the likely presence of a pathogen challenge). The technique of treatment (cold, steam distillation, extraction or maceration with non-aqueous solvents…) has been also reported to change the active substances and related compounds in the final product [22].

Among PFAs, there is a rising interest in EOs for animal nutrition as some of these feed additives have been shown to possess a much higher biological activity compared to the raw material they were extracted from [27]. EOs are complex mixtures of volatile compounds, being mainly hydrocarbons (terpenes, sesquiterpenes), oxygenated compounds (alcohol, aldehydes, ketones) and a small percentage of non-volatile residues (paraffin, wax) [28]. Chemically, EOs consist fundamentally of two classes of compounds, the terpenes and phenylpropenes. Although, the effects of a mixture of EOs rely on the additive and synergy or antagonic effects of their components, 2 or 3 components may constitute up to 85% of the total mixture [29] and thus, contribute to its primary property [2]. In fact, thymol and carvacrol are the two main phenols which account for almost 80% of the EO of oregano and are the main contributors to its antibacterial and antioxidant activities. The compound p-cymene is another dominant component of oregano EO [30]. Even though this component is not considered as an effective antimicrobial agent, it is a precursor for carvacrol which possesses higher preference for liposomal membranes, enabling carvacrol to be more easily transported into the cell [31].

EOs are perceived as growth promoters in poultry diets with strong antimicrobial and anticoccidial activities [2]. Thus, literature shows that growth promoting effects of EOs exist both abundantly and controversially, which makes it imperative to perform more in-depth research to understand the underlying mechanisms.

## 4. Effects of Phytogenic Feed Additives on Chickens

The effects of PFAs on the main metabolic and physiologic process like nutrient digestibility, intestinal microbiota, immunity, oxidant status and growth performance of broilers will be discussed with details below.

### 4.1. Effects of Dietary PFA Supplementation on Growth Performance

Several studies have been carried out using herbs, spices, and EOs and showed inconsistent results on chicken performance. Although some studies showed that PFAs have positive effects on body weight gain and FCR in chickens [32,33], others reported either an improved chicken body weight gain without affecting FCR [34,35] or an enhanced feed conversion rate associated to a lack of effects on body weight or feed intake [36,37]. This inconsistency may be explained by several factors such as the botanical source, the concentration and the duration of supply of the active compounds, the feed composition, and the experimental challenging conditions, animal age and health status [38].

#### 4.1.1. Chickens Maintained under Non-Challenging Conditions

Detailed results of some recent studies evaluating the effects of PFAs on growth performance of birds maintained under non-challenging conditions are presented in Table 1, Table 2 and Table 3. Dietary supplementation of EOs containing menthol, anethol and eugenol [39] as well as carvacrol alone [40], or combined with either thymol [41,42,43] or thymol and limonene [44] has been shown to promote growth performance of broiler chickens. Similar positive effects on production performance were obtained by supplementing laying hens diets with thymol and cinnamaldehyde [39], star anise oil [45], *Citrullus lanatus* EO [46], tea tree EO [47] and peppermint oil [48]. Several other studies have also evaluated the effects of supplementing black cumin (*Nigella sativa* L.) seeds on broilers, quails and laying hens. Although some authors failed to find any effect by supplementing Japanese quails diets with black cumin [49], others reported improved growth performance in quails [50,51] broiler chickens [52] and laying hens [53,54]. These growth-promoting effects have been attributed to the presence of a large number of pharmacologically active compounds such as thymoquinone, dithymoquinone, thymohydroquinone, nigellone, melanthin, nigilline, nigelamine, damascenone, *p*-cymene and pinene and a variety of essential nutrients including vitamins A, B, C, D and E, as well as minerals such as magnesium, calcium, phosphorus, potassium, iron, cobalt, zinc and manganese [41,55]. Moreover, curcuminoids and lipophilic turmeric extract containing curcumin and turmerones; known for their gastroprotective and anti-inflammatory activities, showed positive effects on growth performance of slow-growing [56] and fast-growing broiler chickens [57], respectively. Similar growth-promoting effects were observed by supplementing broiler chickens by a PFA of *Aerva lanata*, *Piper betle*, *Cynodon dactylon*, and *Piper nigrum* [58], *Pulicaria gnaphalodes* powder [59], *Achyranthes japonica* extract [60], *Boswellia serrata* [61] and bioactive olive pomace extract from *Olea europaea* [62] as well as laying hens by fennel seeds or red pepper [53] a mixture of *Punica granatum*, *Thymus vulgaris*, and *Allium sativum* [63] and dietary Nettle (*Urtica cannabina*) [64]. However, egg weight, laying rate and FCR were not improved by dietary supplementation of either EO of star anise (*Illicium verum* Hook.f.) [45] or a mixture of 13.5% thymol and 4.5% cinnamaldehyde [65].

#### 4.1.2. Chickens Maintained under Challenging Conditions

A large number of studies have been performed to investigate the positive effects of PFAs on broiler chickens subjected to challenges related to environmental conditions including heat stress, lipopolysaccharide (LPS) and pathogens such as *Clostridium perfringens, Eimeria* spp, *Salmonella typhimurium*, or *Escherichia coli*, among others. Results of some recent studies are illustrated in Table 2 and Table 4.

Heat stress in chickens induces a tight junction disruption that may lead to increased gut permeability and eventually to a dysregulation of the body’s homeostasis [66], and thereby poor nutrient absorption, increased secretion of electrolytes and water in gastrointestinal tract leading to compromised performance [67]. However, the adverse effects caused by heat stress on broiler growth performance have been shown to be alleviated by the supplementation of turmeric rhizome powder [68], enzymatically treated *Artemisia annua* [69], ginger [70] and curcumin [71]. *In ovo* injection of black cumin (*Nigella sativa*) extract improved post-hatch performance of thermally challenged broiler chickens during incubation [72]. Similarly, 59-week-old cold-stressed laying hens supplemented with oregano EO showed improved FCR and egg production from week 9 to 12 of a 12-week feeding trial [73].

Numerous studies have been also performed to discern the efficacy of PFAs in chickens subjected to coccidiosis and necrotic enteritis (NE) classified among the most significant diseases affecting the poultry industry, which have become more prominent in the wake of policies to reduce the use of antibiotics in animal production. *Clostridium perfringens*-challenged broilers supplemented with PFAs consisting of either benzo [c]phenanthridine alkaloids from *Macleaya cordata*, active component of carvacrol from oregano (4.95 g/100 g), cinnamaldehyde from cinnamon (2.97 g/100 g), and capsaicin from paprika (1.98 g/100 g), or EO of thyme and anise as leading active ingredients and other including oregano, carvacol, yucca extract and cinnamaldehyde exhibited similar growth performance as the control non-infected group [74]. Microcapsules with a blend of EOs (thyme, peppermint, savoury, and black pepper) at the dose of 0.5, 1, and 2 kg/ton in the *C. perfringens*-challenged broiler chickens results in raising final weight and total feed intake [75]. However, supplementation of EO containing 25% thymol and 25% carvacrol as active components, did not influence the growth performance during d 0 to 14 and tended to linearly reduce the FCR between 14 and 28 d of age of *Clostridium perfringens* challenged broilers [76]. Regarding coccidiosis, the supplementation of a cashew nut shell oil and commercial castor oil blend allowed a recovery in performance similar to that observed with birds receiving the ionophore monensin during the accumulated experimental period (1 to 42 d) [77]. Hussein et al. reported that combinations of peppermint, chamomile and prebiotic yeast cell wall were as effective as salinomycin in preventing the decline in the weight gain and FCR performance of coccidiosis-challenged broilers [78]. However, the effects of dietary *Yucca*-derived saponin supplementation on growth performance seem to be dependent on broiler health status. Although Su et al. [79] reported that saponin supplementation via an extract from *Y. schidigera* serves as an effective growth promoter in non-challenged broilers, Oelshlager et al. [80] found no significant influence of *Yucca* extract on growth responses of broilers during a mixed coccidian challenge. This suggests a reduction in the bioefficacy of saponins when used during an immune challenge [80].

Similarly, a 20-day experiment showed that dietary curcumin supplementation from day 12 to 20 failed to positively affect growth performance of broiler chickens challenged with *Eimeria* species on day 14 of age [81]. These authors attributed the lack of effect to the short period of supplementation and suggested that it might be beneficial if curcumin is fed in broilers for a 42-d period. This suggestion was based on the results of Rajput et al. [82], showing that birds fed curcumin-supplemented diets for 42 d exhibited significant increase in the BW and feed efficiency during the finisher stage (22–42 d), whereas no significant difference in growth performance was observed during the starter phase (0–21 d).

PFAs supplementation has been also shown to alleviate the effects on the broilers growth performance of other pathogens, such as *Achyranthes bidentate* under *Escherichia coli* challenge [83], resveratrol under *Escherichia coli* challenge [84], a PFA consisting of various nutritional acids and four different alkaloids obtained from special plants under *Salmonella typhimurium* challenge [85], and *Allium hookeri* roots in LPS-induced young broiler chickens.

**Table 1:** Effects of dietary supplementation of PFAs on growth performance of broilers under non-challenging conditions.

Feed Additive	Major Components	Dose, (mg/kg Diet)	Diet	Age	Treatment Effects (%, Compared to Control)			References
					BW	ADFI/FI	FCR	
*Olea europaea* extract	Triterpenes (10%) polyphenols (2%)	750	Wheat-soybean meal based diet	21–42 d	NM	NS	−7.9	[62]
*Achyranthes japonica* extract	Flavonoid (1.15 mg/g), polyphenol (4.26 mg/g) and saponin (0.47 mg/g)	1000	Corn-soybean meal based diet	0–35 d	3.5	−2.4	−6.2	[60]
EOs	Carvacrol (20%) and thymol (25%)	200	Corn-soybean meal based diet	29–42 d	NS	−9.6	−11.8	[43]
EOs	Carvacrol (5%), cinnamaldehyde (3%), and capsicum oleoresin (2%)	100	Corn-soybean or wheat -soybean meal based diet		16.4	6.1	−9.4	[86]
*Aerva lanata*, *Cynodon dactylon*, *Piper nigrum* and *Piper betle*	Phenolic acid contents (10,176.8 μg/g), flavonoids (53.0 μg/g), other (220.2 μg/g)	10,000	Corn-soybean meal based diet	0–42 d	14.1	NS	−14.0	[58]
*Pulicaria gnaphalodes* powder	Phenolic compounds, alkaloids, terpenoids, and triterpene saponins	3000	Corn-soybean meal based diet	0–42 d	4.3	NS	−3.0	[59]
Standardized lipophilic turmeric extract	3.1% of curcuminoids content and terpenes (turmerones)	10,000	NM	0–42 d	9.0	1.6	−7.7	[57]
EOs	Carvacrol (63.5%), thymol (3.4%) and paracymene (13.1%)	400 μL	Corn-wheat-soybean meal based diet	28–43 d	4.2	NS	−3.9	[40]
Thyme powder	Major EO (thymol (50.48%), γ-terpinene (11.03%), P-cymene (9.77%), and carvacrol (4.30%)), phenolic acids (salicylic acid (2450.03 ppm), ellagic acid (1240.42 ppm)) and flavonoid compounds	5000	Corn-soybean meal-based diet	0–42 d	4.6	3.3	NS	[87]
EOs (oregano, anise, and citrus peel; CBP)	Carvacrol: 102 g of the chemical component/kg of CBP	150	Corn-soybean meal-based diet	0–42 d	NS	-5.3	NS	[88]
Combination of herbs, spices, EOs and extracts	Mainly EOs from mint, star anise and cloves	100	Corn-soybean meal-based diet	0–42 d	7.0	NS	NS	[39]
EO (powdered and matrix-encapsulated form)	-Powdered: menthol and anethole -Encapsulaed: carvacrol, thymol, and limonene	150 100	Corn-wheat-soybean meal based diet	0–42 d	NS 2.4	NS NS	NS NS	[44]
EOs	Oregano containing carvacrol (26.4 mg/kg) or thymol (13 mg/kg)	300 600	Corn-soybean meal-based diet	0–42 d	7.8 9.6	4 8	NS NS	[41]
Spices: *Nigella sativa* seeds	Thymoquinone, dithymo- quinone, thymohydroquinone, nigellone, melanthin, nigilline, nigelamine, damascenone, *p*-cymene and pinene	10,000 20,000	Corn-soybean meal-based diet	0–35 d	3	NS	5.6	[52]

NS: not significant; NM: not mentioned.

**Table 2:** Effects of dietary supplementation of PFAs on growth performance of laying hens.

Feed Additive	Major Components	Dose (mg/kg Diet)	Diet	Line and Age	Main Findings	References
Non-Challenging Conditions						
*Mentha arvensis* (MA) and *Geranium thunbergii* (GT) extracts	MA: menthol, isomenthol, neomenthol, p-cymene, d-menthone, eugenol, and cineol GT: citronellol, isomenthone, and geraniin	100, 500 and 1000	Corn-wheat- soybean meal based diet	Hy-Line Brown layers (28–44 weeks)	↑ FI, egg production and egg weight	[89]
Fermented pine (*Pinus densiflora*) needle extract	α-pinene, caryophyllene, beta-pinene and bisbenzene, camphene, borneol, phellandrene, quercetin, kaempferol, and terpene	2.5 and 5	Corn-soybean meal-based diet	Hy-Line Brown laying hens (40–46 weeks)	↑ FI, egg production and egg mass	[90]
Fermented *Schisandra chinensis* pomace (SC), fermented *Pinus densiflora* (PD) needle extract, and *Allium tuberosum (AT)* powder	SC: lignin PD: phenolics, flavonoids, and tannins AT: organosulfur compounds, polyphenols, and saponins	1000 and 3000	Corn-soybean meal-based diet	Hy-line brown laying hens (48–54 weeks)	-=Egg production, daily egg mass and FCR. -↑ FI	[91]
Dry leaf extract of peppermint (*Mentha piperita L*.)	Menthol	0, 74, 148, 222, and 296	Corn-soybean meal-based diet	Bovans Brown laying hens (32–44 weeks)	↑ FI, egg production, egg weight and egg mass	[48]
*Citrullus lanatus* EOs	Phenolics (1.57 mg/100 g) Sterols (600.56 mg/100 g) Flavonoids (163.5 mg RE/kg)	1000 and 2000	Corn-soybean meal-based diet	White Leghorn laying hens (18–26 weeks)	↑ Weight gain, ADFI, ADG and egg mass; ↓ FCR	[46]
Tea tree (*Melaleuca alternifolia)* EO	Terpinen-4-ol (40.0%), γ-Terpinene (23.0%) and α-Terpinene (10.4%)	40 and 80	NM	Lohmann Brown hens (55–58 weeks)	↑ Daily egg production and ↓ FCR	[47]
Thyme *(Thymbra spicata*) and Rosemary (*Rosemarinus officinalis*)	-Thyme: Carvacrol (87.81%), thymol (9.58%), L-Linalool (0.86%), borneol (0.74%) -Rosemary: 1.8 cineole (34.08%), camphor (27.95%), alpha-Pinene (14.50%), borneol l(8.65%), alpha-Terpineol (7.39%), alpha-Thujone (1.09%), camphene (0.55%)	1000 for each source	Corn-soybean meal-based diet	Bovans-White (48–56 weeks)	-No effects on FCR -↓ Egg production and egg weight	[92]
Cumin (*Cuminum cyminum L*.) seed oil	Cuminol, cuminique alcohol, cuminaldehyde, cymine, phellandrene, carvone, cymol, terpenes, α-pinene…	500	Corn-soybean meal-based diet	Boven hens (24–30 weeks)	=Egg production rate, egg mass and FI ↓ FCR and ↑ egg weight	[93]
Eucalyptus leaves	Polyphenols	500, 800 and 1200	Corn-soybean meal-based diet	Yueqinhuang laying hens (35–44 weeks)	↑ Egg production and egg mass	[94]
Fennel seeds (F), black cumin (BC) seeds and hot red pepper (RP)	F: trans-anethole BC: thymoquinone, anethole, carvacrol and 4-terpinol RP: Capsaicin	5000 for each	Corn-soybean meal-based diet	Lohmann Brown Lite laying hens (32–40 weeks)	↑ Egg weight, egg production, egg mass and ↓ FCR by F and RP	[53]
Green tea	Polyphenols	200	Corn-soybean meal-based diet	Hy-line Brown (65–74 weeks)	↑ Egg production and ↓ FCR	[95]
EOs	Thymol (13.5%) and cinnamaldehyde (4.5%)	50, 100 and 150	Corn-wheat-soybean meal based diet	Lohmann White (54–65 weeks)	=Egg production, egg weight, egg quality, FI and FCR	[65]
*Echinacea purpurea* powder	Caffeic acid and alkamids, phenolic acids, polyacetylenes	2500, 5000, 7500 and 10,000	Corn-soybean meal-based diet	Leghorn laying hens (43–53 weeks)	↑ Egg production and egg mass	[96]
Peppermint EO Thyme EO	-Menthol and menthone -Thymol, γ-Terpinen and ρ-Cymene	1000	Corn-soybean meal-based diet	Lohmann LSL-lite (40–48 weeks)	↑ Egg production and egg mass ↓ FCR	[97]
Dried grape pomace	Polyphenols	40,000 and 60,000	Corn-soybean meal-based diet	Bovans laying hens (80–92 weeks)	=Live weight, feed intake, egg production and feed efficiency	[98]
Fennel (F) and thyme (T) extracts	F: anethole, limonene T:-Thymol, γ-Terpinen and ρ-Cymene	40	Corn-soybean meal-based diet	Hy-Line White (26–38 weeks)	↑ Egg weight and egg mass	[99]
Cold stress + *Escherichia* *coli*						
*Curcuma longa*	Curcumin	200	Corn-soybean meal-based diet	Hy-Line Brown laying hens (84–90)	=Egg production, egg mass, feed intake and FCR	[100]
Cold stress						
Oregano EO	Carvacrol and thymol	50, 100, 150 and 200	Corn-soybean meal-based diet	Semi-heavy laying hens (59–71 weeks)	=FCR, egg production and egg mass	[73]
Heat stress						
Grape pomace flour	Polyphenols	10,000, 20,000 and 30,000	Corn-soybean meal-based diet	Hy-Line lineage (74–79 weeks)	↑ FI	[101]

ADG: Average Daily Gain; ADFI: Average Daily Feed Intake; FI: Feed Intake; FCR: Feed Conversion Ratio; ↑: increased; ↓: decreased; =: equal.

**Table 3:** Effects of dietary supplementation of PFAs on growth performance of other birds under non-challenging conditions.

Feed Additive	Major Components	Dose (mg/kg Diet)	Diet	Line and Age	Main Findings	References
Grape seed extract	Polyphenols	100 and 200	Corn-soybean meal-based diet	Duckling (Pekin- female; 0–6 weeks)	↑ ADG, and final body weight with ↓ FCR	[102]
Oregano EO	Carvacrol and thymol (85%)	100	Corn-soybean meal-based diet	Duckling (Cherry valley; 0–5 weeks)	=ADG, FCR	[103]
Eucalyptus (*Eucalyptus camaldulensis*)	p-cymene, 1, 8-cineole, b-phellandrene, spathulenol, cryptone aldehydes, cuminal, phellandral, and a-phellandrene	100 and 200	NM	Laying Japanese quails	=Productive traits	[104]
Oregano EO	Thymol (5%) and carvacrol (65%)	150 and 300	Corn-soybean meal-based diet	Duckling (Cherry valley; 11–42 days)	=Final body weight, ADG, FI, and FCR	[105]
NM	Thymol	2000, 4000 and 6250	NM	Quail (*Coturnix japonica*; 85–128 days)	=BWG, FI, egg production, and egg weight	[106]
Leaves of *Astragalus membranaceus*	Polyphenols (saponins, flavonoids)	10,000, 30,000 and 50,000	Corn-soybean meal-based diet	Japanese quail (0–35 days)	↑ FI, and weight gain	[107]
*Mentha piperita* (peppermint)	Phenolic compounds	10,000, 20,000, 30,000 and 40,000	Corn-soybean meal-based diet	Quail	=FI and ADG	[108]

BWG: body weight gain; ADG: Average Daily Gain; ADFI: Average Daily Feed Intake; FI: Feed Intake; FCR: Feed Conversion Ratio; ↑: increased; ↓: decreased; =: equal.

**Table 4:** Effects of dietary supplementation of PFAs on growth performance of broilers under challenging conditions.

Feed Additive	Major Components	Dose, (mg/kg Diet)	Diet	Age	Treatment Effects (%, Compared to Control)			References
					BW	ADFI/FI	FCR	
*Clostridium perfringens*								
Herb: *Macleaya cordata* plant	Four specific alkaloids mainly sanguinarine and protopine	120	Corn-soybean meal-based diet	15–35 d	12.7	NS	−14.8	[109]
Plant: *Macleaya cordata* Plant extracts EOs	Benzo [c]phenanthridine alkaloids Carvacrol (4.95 g/100 g), cinnamaldehyde (2.97 g/100 g), and capsaicin (1.98 g/100 g) Thyme and anise, oregano, carvacol, yucca extract and cinnamaldehyde	NM NM NM	Corn-soybean meal-based diet	15–21 d	NS NS NS	NS NS NS	−8.9 −10.0 −11.6	[74]
EO	Thymol (25%) and carvacrol (25%) as active components	60, 120 and 240	Wheat-soybean meal-based diet	14–28 d	NS	NS	NS	[76]
*Eimeria*								
Herb: *Curcuma longa*	Curcumin	100 and 200	Corn-soybean meal-based diet	12–20 d	NS	NS	NS	[81]
EOs: cashew nut shell liquid and castor oil	Cardanol, cardol, and anacardic acid Ricinoleic acid	1500	Corn-soybean meal-based diet	0–42 d	2.3	NS	NS	[77]
*Escherichia coli*								
Resveratrol	Polyphenols	600	Corn-soybean meal-based diet	0–42 d	6.1	2.2	−3.9	[84]
*Salmonella typhimurium*								
Plant: *Macleaya cordata*	Benzo [c]phenanthridine alkaloids	5000	Corn-soybean meal-based diet	8–15 d	NS	NS	−11.0	[85]
Heat-Stress								
Plant: Turmeric	Curcumin	100	Corn-soybean meal-based diet	21–42 d	NS	NS	−2.8	[71]
Herb: *Zingiber officinale*	Gingerdiol, gingerol, gingerdione, and shogaols	2000	Corn-based diet	0–42 d	3.3	NS	3.0	[70]
Herb: *Artemisia annua*	Phenolics (44.24 mg GAE/g) and flavonoids (27.8 mg RE/g)	1000	NM	21–42 d	8.2	4.1	NS	[69]
*Turmeric rhizome* powder	Phenolic compounds: curcuminoids	2000	Corn-soybean meal-based diet	0–42 d	10.6	NS	6.9	[68]

GAE: Total phenolic contents were expressed as Gallic Acid Equivalents (mg GAE/g); RE: total flavonoid content was expressed as Rutin Equivalents (mg RE/g); NM: Not Mentioned; NS: Not Significant; BW: Body Weight; ADFI: Average Daily Feed Intake; FI: Feed Intake; FCR: Feed Conversion Ratio.

### 4.2. Effects of Dietary PFA Supplementation on Digestibility

Evaluating digestibility is important as it directly contributes to the animal feed efficiency. However, improving the digestibility is crucial not only for better feed efficiency but also to reduce the amount of undigested feed in the gut, which may favour the occurrence of intestinal imbalances. These imbalances may lead to inflammatory processes and accelerated turnover of intestinal tissue, which results in poorer performance.

Numerous studies have been carried out to study the effects of PFAs inclusion on nutrient digestibility in broiler chickens, laying hens as well as ducks and showed inconsistent results.

The use of either extracts from olive leaves rich in polyphenols [110] or a bioactive olive pomace extract from *Olea europaea* [62] failed to enhance nutrient apparent total tract digestibility (ATTD) coefficients, or apparent ileal digestibility (AID) of dry matter (DM), organic matter (OM), ether extract (EE), and gross energy (GE), respectively. However, the use of EOs such as a blend of carvacrol combined with either cinnamaldehyde and capsicum oleoresin [111] or thymol and limonene in encapsulated forms [44] increased fat digestibility (FD), the AID of crude protein (CP), phosphorus and cysteine. Similarly, a combination of over 30 essential oils and phytogenic compounds increased the digestibility of DM, CP and EE [112].

In laying hens, dietary supplementation of peppermint oil at 0, 74, 148, 222, and 296 mg/kg linearly increased digestibility of CP, EE, and phosphorus from 32 to 44 weeks of age [48], while 100 mg/kg of EOs including thymol 13.5% and cinnamaldehyde 4.5% as major active components significantly increased protein and fat digestibility from 54 to 65 weeks of age [65].

An increased nutrient digestibility was also obtained by supplementing meat-type ducks fed high nutrient density diets by a phytogenic blend containing quillaja, anise, and thyme [113]. Similar results were obtained in broiler chickens supplemented with an EO blend, a quillaja saponin blend, or a combination of both phytogenic preparations [114] or a PFA of *Aerva lanata*, *Piper betle*, *Cynodon dactylon*, and *Piper nigrum* [58]. These authors attributed the digestion-stimulating properties of the PFA basically to piperine, which has been previously reported to stimulate digestion and increase absorption of selenium, vitamin B complex, β carotene, and other nutrients.

Possible mechanisms behind improved nutrient digestibility by PFAs supplementation could be attributed to the ability of these feed additives to stimulate appetite, saliva secretion, intestinal mucus production, bile acid secretion, and activity of digestive enzymes such as trypsin and amylase as well as to positively affect the intestinal morphology [58] or to possess an overlapping mode of action including local effects at the intestinal border and systemic alterations of macronutrient metabolism by these feed additives [114].

### 4.3. Effects of Dietary PFA Supplementation on Intestinal Microbiota

It is well-known that farm animal performance is directly linked with gut function and health, which is determined by the continuous interaction among diet, intestinal integrity, gut microbiome and the immune system of chickens. Keeping in view the importance of intestinal microbiome [115], several authors studied the effects of PFAs on the microbiota composition (Table 5). The results showed that these effects are both phytogenic composition and inclusion level dependent [116,117], and can range from neutral with no effect [117,118,119] up to beneficial [120,121,122,123,124,125].

The supplementation of a PFA containing the carvacrol as main active compound [124] was shown to modulate the intestinal microbiota more at cecal rather than ileal level by increasing cecal mucosa-associated levels of *Bacteroides* spp., *Clostridium cluster IV, and Clostridium cluster XIVa*. This increase could be considered beneficial as Clostridia are not only dominant in ceca [126], but they also contribute to the maintenance of overall gut function, especially through butyrate production [127]. *Deinococcus*, Bacillaceae and Caulobacteriales were increased in ileal digesta of laying hens fed on a dietary supplementation of EOs, promoting an increase of digestive enzyme activity leading to improved feed utilization efficiency [128]. Moreover, although some authors reported a decrease of the relative abundance of beneficial commensal bacteria such as *Lactobacillus* by plant extract supplementation [43,129], several others found Clostridiales and/or Lactobacillales to be higher in broilers supplemented with EOs of carvacrol and thymol [130,131] or thymol, eugenol and piperine [121]. More precisely, addition of thymol and carvacrol to the diet changes the host ileum microbial population dynamics by increasing the abundance of *L. crispatus* and *L. agilis*, and decreasing *L. salivarius* and *L. johnsonii* [131]. *L. crispatus* is known to be a rod-shaped species of the genus *Lactobacillus* and is a hydrogen peroxide-producing beneficial microbial species that plays a key role in the protection of the host from infection [132]. A study conducted by Gudiña et al. [133] revealed that a biosurfactant produced by a *L. agilis* strain showed considerable anti-adhesive activity against *S. aureus*, as well as antimicrobial activity against *P. aeruginosa, S. aureus* and *S. agalactiae*. Green tea and pomegranate have also been proven to modulate the intestinal microbiota [122,134], especially by promoting beneficial bacteria in the intestinal tract [125,135]. In a recent study conducted by Perricone et al. [123], broilers receiving a plant extract composed of green tea leaves (*Camellia sinensis*) and pomegranate rinds (*Punica granatum*) promoted greater relative abundance of lactic acid bacteria compared to the control group. These results are of particular interest because lactic acid bacteria are recognized to positively affect the intestine by regulating the composition of intestinal microflora, developing intestinal immunity and promoting gut health [136].

**Table 5:** Effects of dietary PFA supplementation on microbiota of broilers.

Feed Additive	Major Components	Dose	Duration of Supplementation	Site and Age of Sampling	Main Effects on Microbiota	References
Green tea leaves (*Camellia sinensis*) and pomegranate rinds (*Punica granatum*)	Green tea: catechins Pomegranate: tannins and flavonoids	2 mL/L in drinking water	From 0 to 4 days, 10, 11, 20, and 21.	Cecum Day 50	Family: ↑ Lactobacillaceae and Peptococcaceae Genus: ↑ *Roseburia* and ↓ *Shuttleworthia*	[123]
*Aerva lanata*, *Cynodon dactylon*, and *Piper nigrum* (2 kg from each) and *Piper betle* (2 L.)	Phenolic acid contents (10,176.8 μg/g), Flavonoids (53.0 μg/g), others (220.2 μg/g)	1 and 2% in the feed	42 days	Cecum 42 days	↑ *Bifidobacterium*	[58]
EOs	Carvacrol (102 g/kg PFA)	115 g/kg in the feed	42 days	Ileum and cecum 42 days	↑ Cecal Bacteroides, Clostridium cluster IV, and Clostridium cluster XIVa	[124]
EOs	Carvacrol (20%) and thymol (25%)	200 (LPE) and 400 (HPE) g/mg in the feed	42 days	Duodenum, ileum, and cecum 14 and 28 days	-Day 14: ↑ Firmicutes, Bacteroidetes and Thermi in the ileal microbiota of the HPE group ↓ Proteobacteria and Tenericutes, and 10 genera (e.g., Ruminococcus, Faecalibacterium) -Day 28: ↑ Bacteroidetes and Cyanobacteria and three genera (e.g., Alistipes) in the cecal microbiota of the HPE group ↓ Actinobacteria and two genera (Lactobacillus and unclassified Coriobacteriaceae).	[43]
-Oregano essential oil (OEO) -Commercial blend of phytogenic (CBP)	5% essential oil of *Origanum vulgare subsp. Hirtum* plants -Carvacrol (102 g/kg CBP)	300 and 500 ppm 150 ppm in the feed	42 days	Ileum 21 days	↓ *Escherichia* *coli* for both OEO and CBP groups compared to the NC. *=Lactobacillus*	[137]
EOs	Thymol, eugenol and piperine (29%)	0.03% in the feed	35 days	Ileum 35 days	↑ *Lactobacillus* counts ↓ *Escherichia* *coli* counts	[121]
Tea polyphenols (TP)	Caffeine (69.8 mg), (–)-EGCG (495 mg), (–)-epicatechin gallate (112 mg), (–)-epicatechin (100 mg), (–)-epigallocatechin (78 mg) and (–)-gallocatechin gallate (96 mg/1000 mg TP)	0.03, 0.06 and 0.09 kg^−1^ BW in the feed	56 days	Ileum mixed with cecum 56 days	↑ Species of *Lactobacillus reuteri*, uncultured *Bacteroides* sp. and *L. crispatus*	[135]
EOs	Thymol (25%) and carvacrol (25%)	120 mg/kg in the feed	21 days	Ileum 21 days	↑ *Lactobacillus crispatus* and *Lactobacillus agilis* abundance ↓ *Lactobacillus salivarius* and *Lactobacillus johnsonii* abundance	[131]
EOs	Equal mixture of thymol plus carvacrol	100 and 200 mg/kg in the feed	42 days	Duodenum, jejunum, and ileum; 24 days	↑ *Lactobacilli* counts ↓ *Escherichia* *coli* and *Clostridium perfringens* counts with 200 mg/kg	[120]
EOs	Thymol (25%) and carvacrol (25%)	60, 120, and 240 mg/kg in the feed	28 days	Ileum and cecum; 21 and 28 days	Ileum: ↓ *Escherichia* populations Cecum: ↓ numbers of total bacteria and Escherichia on day 28	[130]

↑: increased; ↓: decreased; =: equal.

### 4.4. Effects of Dietary PFA Supplementation on Immunity

An increasing number of studies have shown that health-promoting activities of phytochemicals are attributed to their ability to improve host defence against microbial infection [138]. The immune activating properties of several phytochemicals such as dandelion (*Taraxacum officinale*), mustard (*Brassica juncea*) and safflower (*Carthamus tinctorius*) [139] as well as thistle (*Silybum marianum*), turmeric (*Curcuma longa*), reishi mushroom (*Ganoderma lucidum*), and shiitake mushroom (*Lentinus edodes*) [140] have been evaluated in vitro using avian lymphocytes and macrophages. In both studies, all extracts inhibited tumour cell growth and stimulated the innate immunity in poultry. These results were further confirmed by several in vivo trials, which are giving increasing evidence that through interactions with immune system, PFAs can modulate immune responses through various mechanisms. Some of the most recent studies are synthesized in Table 6. Dietary immunomodulation is a key to enhance the productivity and immune system integrity of farm animals raised under the absence of antibiotics [141]. One of those mechanisms is the modulation of the expression of the cytokines playing a key role in both the adaptive and the innate immune system [142,143].

A study conducted by Pirgozliev et al. [86] demonstrated that broilers under non-challenging conditions, supplemented by a commercial blend comprising 5% carvacrol, 3% cinnamaldehyde, and 2% capsicum oleoresin showed downregulated IFN-γ and IL-6 cytokines indicating a lower inflammation level than those in the other groups.

In broilers under challenging conditions such as necrotic enteritis, Lee et al. [150] showed that a mixture of *Capsicum* and turmeric oleoresins reduced intestinal IL-8, lipopolysaccharide-induced TNF-α factor (LITAF), IL-17A and IL-17F mRNA levels. Similarly, the expression of pro-inflammatory cytokines was reduced by the supplementation of *Allium hookeri* in LPS-induced young broiler chickens [148] and thyme powder in broilers without any challenge [87]. Nonetheless, a blend of cashew nut shell liquid (CNSL) and castor oil modulated the inflammatory response of broiler chickens against *Eimeria* spp. by increasing gene expression of TNF-α, IL-6 and IFN-γ and reducing expression of IL-1 and COX-2, one week post-infection [146] These authors concluded that although inflammation is a highly undesirable phenomenon owing to its costly effects on animal production efficiency, this increased inflammatory response observed in challenged birds treated with CNSL–castor oil was necessary to help the immune system to effectively fight against coccidiosis and other pathogenic bacteria to prevent intestinal dysbiosis.

Other authors have rather evaluated the levels of SIgA, IgM, and IgG, three major classes of immunoglobulin in chickens that play key roles in the maintenance of immunity [151]. SIgA is involved in the protection and homeostatic regulation of intestinal mucosal epithelia by limiting the access of numerous microorganisms and mucosal antigens, while IgG directly contributes to an immune response including neutralization of toxins and viruses. A decrease of SIgA and IgG observed in the jejunum mucosa of heat-stressed broilers was counteracted by the supplementation of enzymatically treated *Artemisia annua* [69]. Similarly, an increase of IgG was observed by the supplementation of thyme powder [87] and EOs of oregano [88] in broilers raised under normal conditions or by the supplementation of resveratrol in chickens challenged with *Escherichia coli* [84].

The effect of PFAs, especially EOs, on the immune response to some viral vaccines in broiler chickens was also evaluated in several studies. A mixture containing oregano oil (50 g), carvacrol (10 g), thyme oil (33.33 g), eucalyptus oil (50 g), thymol (5 g), eucalyptol (10 g), and acacia (Arabic gum) surfactant (27 g) supplemented through the drinking water at a dose of 0.5 mL/L showed an immune-stimulating response to Newcastle disease (ND) and infectious bursal disease (IBD) vaccines, antiviral effect against ND virus, especially if administered before the challenge [145].

The supplementation of cinnamon bark oil (CNO) [147,152], clove bud oil (CLO) and ajwain seed oil (AJO) [147], cinnamaldehyde combined with formic acid [147] and Origanum essential oil containing 60.2% carvacrol and 4% thymol [149] enhanced immune response against NDV in broiler chickens. However, antibody titres against avian influenza virus and NDV were unaffected by cinnamon bark powder supplementation (2 and 4 g/kg diet) [153] or clove EO (0.15, 0.30 and 0.45 g/kg) [154], and antibody titres against NDV vaccine and sheep red blood cells were not significantly influenced by the supplementation of EOs mixture obtained from anise, oregano, and citrus peel in broiler chickens [155].

The supplementation of black cumin (*Nigella sativa*) combined with *Echinacea* enhanced the immune response after AI-H9N2 vaccination and reduced the pathogenicity of infection in dexamethasone-stressed chickens [156]. The immune-modulating effects of black cumin may be attributed to pharmacologically active constituents, such as thymol, nigellidine, nigellimine, thymoquinone, dithymoquinone and thymohydroquinone [157] which are able to induce pharmacological effects against antigenic challenge [158].

### 4.5. Effects of Dietary PFA Supplementation on Blood Biochemical Parameters and Oxidant Status

Several authors studied the effect of PFA supplementation on the serum biochemical indicators that help to display the nutrient’s metabolism and body physiological state [159]. Serum lipid parameters have been shown to be reduced in broiler chickens by the supplementation of lavender EO (cholesterol and LDL-C; [160]), *Pulicaria gnaphalodes* powder (cholesterol and triglycerides; [59]) and a mixture of oregano, anise and citrus EOs (cholesterol; [161]). Serum cholesterol has been also reduced in laying hens supplemented by EOs [162], either black cumin or hot red pepper [53], peppermint oil [48] or nettle *Urtica cannabina* [64].

The mechanisms explaining the hypocholesterolemic effect of PFAs may be associated to the reduced activity of enzymes involved in lipid metabolism including 3-hydroxy-3-methylglutaryl-CoA (HMG-CoA) reductase (enzyme associated with cholesterol synthesis), cholesterol-7 hydroxylase fatty acid synthase and pentose phosphate pathway [147,163].

The supplementation of PFAs has been also shown to improve the antioxidant status of broilers [164,165,166]. A recent study performed by Paraskeuas et al. [39] revealed that a mixture of menthol, anethol and eugenol increased blood plasma total antioxidant capacity (TAC) in a linear pattern, corroborating the results previously obtained by other authors [167,168]. The activity of superoxide dismutase (SOD) and glutathione peroxidase (GSH-Px), enzymes considered as one of the defensive mechanism of the body against the oxidative stress [169], was increased by supplementing broilers by lavender EO [160] and laying hens by star anise oil [45] and grape pomace [101]. Malondialdehyde (MDA) levels were also decreased in laying hens supplemented by grape pomace [98] and star anise oil [45].

### 4.6. Effects of Dietary PFA Supplementation on Meat, Internal and External Egg Quality

Benefits of dietary PFA supplementation on the quality and shelf-life of meat products are still ambiguous. Although the supplementation of a PFA based on EOs of carvacrol, thymol and cinnamic aldehyde was unable to prevent broiler meat lipid peroxidation caused by freezing temperatures [42], other authors reported increased antioxidant levels and reduction of lipid peroxidation by *Nigella sativa* seeds [52], turmeric [57], curcuminoids [56] as well as herbal components containing curcumin, carvacrol, thymol and cinnamaldehyde [144]. Manipulating lipid peroxidation has been shown to be, in part, achieved through modulating the profile of meat fatty acids by PFAs supplementation. Total saturated fatty acid (SFA) levels were reduced and monounsaturated/polyunsaturated fatty acid (MUFA/PUFA) levels were increased by PFAs supplementation [56,144,170,171]. Particularly, SFA such as lauric, stearic, myristic and palmitic acid are undesirable due to their hypercholesterolemic properties in the form of LDL [144,170]. On the contrary, omega 3 and 6 fatty acids play key roles in human nutrition¸ being precursors of principal molecules involved in the regulation of the cardiovascular and immune system including eicosanoids, prostaglandins, leukotrienes and thromboxanes [144].

External and internal egg quality has been also shown to be influenced by PFAs supplementation. Eggshell thickness was increased by supplementation of herbal EOs mixture [162], peppermint oil [48] and nettle *Urtica cannabina* [64]. A possible mechanism behind these positive effects is the ability of PFAs such as EOs to improve uterine health and increase calcium storage as well as pancreatic secretions, resulting in the enhancement of nutrient digestion and consequently the improvement in eggshell and egg quality [48].

Regarding internal quality, the supplementation of PFAs such as black cumin or red pepper [53], star anise (*Illicium verum* Hook.f.) EO [45] grape pomace flour [101] and curcumin [100] has been shown to reduce egg lipid peroxidation and increase its antioxidant levels, thereby, generating internal stability of the stored eggs and contributing to extending egg shelf life. The Haugh unit score, known as an indicator of egg freshness and is related to shelf life, has been also shown to be increased by PFAs supplementation [46,48,63]. Similar to meat, egg yolk cholesterol content has been shown to be reduced by PFAs supplementation [48,64]. Although the egg yolks of laying hens supplemented with *Citrullus lanatus* EOs showed increased total PUFA and n-6 fatty acids and reduced n-3 fatty acids [46], nettle *Urtica cannabina* supplementation increased total n-3 PUFA concentration while reducing the ratio of n-6/n-3 [64].

## 5. Challenges and Prospective of Using PFAs in Animal Nutrition

### 5.1. Challenges of Using PFAs in Animal Nutrition

Performing systematic and comprehensive studies evaluating the efficacy and safety of PFAs is still difficult due to their complex composition [172]. In addition, inconsistency in the obtained results may be attributed to several factors related either to the enormous variability per se of PFA, including source and bioactive compounds of the PFAs which may depend on the plant, botanical origin growing locations, manufacturing methods, the storage conditions, and the effective dose; or to the environmental conditions, management and rearing conditions (challenge vs. no challenge and differences in the underlying microbial challenge if applied, age, genetics… [22,172]. Some authors also reported that the appropriate minimum inhibitory concentration (MIC) for most phytogenic compounds is higher than the level considered as cost-effective [172]. On the other hand, various phytogenic compounds such as EOs can evaporate rapidly due to their volatile and reactive nature, resulting in largely varied concentrations in the final feed additive. Their effectiveness in animals may also be affected by several conditions during production processes and storage. Thus, ensuring their stability presents a difficulty, as do maintaining their biological activity and masking their strong odour [173]. Moreover, mutual interactions with other substance from feed matrix have been reported such as lower biological effects of PFA present in fibrous diets or high protein diets [174]. Several phytogenic compounds have been also shown to be largely absorbed in the upper GIT, meaning that without proper protection, the majority would not reach the lower gut where they would exert their major functions. In this context, a study conducted by Hafeez et al. [44] showed that the benefits of supplementing the broiler diet with a mixture of encapsulated EOs were higher than the tested PFA in powdered, non-protected form.

Therefore, novel delivery technologies have been developed to protect PFAs from the degradation and oxidation process during feed processing and storage, ease the handling, allow a slower release and target the lower GIT [173]. Among these techniques, microencapsulation is gaining an increasing interest where various carrier types including polymer-based particles such as polysaccharide-protein carriers and lipid-based particles such as vegetable oils and liposomes were tested. The advantages and disadvantages of both carrier materials from the encapsulation efficiency, loading capacity, and release kinetics viewpoints were previously discussed in the literature. Although advantages of polysaccharide-protein carriers include their mechanical and thermal stabilities, nutritional quality, low cost, and easy preparation procedure, they present low encapsulation efficiency, loading capacity, and release efficiency in small intestine [175]. As for lipid-based particles, they are characterized by high encapsulation efficiency, loading capacity, and release efficiency in the small intestine. However, their disadvantages include low mechanical and thermal stabilities [173]. Moreover, liposomes cannot be used for large scale production owing to complex preparation procedures, reduced production capacity, and higher cost [176].

Despite some studies showed some positive effects on chickens by using single EO, several others have rather chosen the combination of various EOs and their isolated components to take advantages from their synergistic effects [2,177]. Indeed, synergistic interactions are of great importance because they enhance the antimicrobial and antioxidant activity by maximizing the efficiencies of the combined agents in the best possible manner which results in several fold reduction in the required doses of EOs applied in situ and thereby lowering their organoleptic impacts. Interestingly, blends containing hydrocarbons and phenylpropanoids (e.g., cinnamaldehyde, eugenol, carvacrol, and capsicum oleoresin) in combination with other components were reported to enhance the bioactivities of these mixtures [178]. A special attention was placed on the interaction of phenolic monoterpenes (thymol, carvacrol) and phenylpropanoids (eugenol) with other groups of components, particularly with other phenols, phenylpropanoids and monoterpenes alcohols, whereas monoterpenes and sesquiterpenes hydrocarbons were used to a lesser extent [178]. Moreover, combining phenolics with monoterpenes alcohols has been reported to produce synergistic effects on several microorganisms, in particular, the combination of phenolics (thymol with carvacrol, and both components with eugenol) were synergistically active against *Escherichia coli* strains [178]. In ruminants, these combinations were the most effective dietary supplementation options that showed ruminal antimicrobial advantages to modulate the ruminal fermentation pathways [179,180].

### 5.2. Prospective of PFAs in Animal Nutrition: Combination of EOs with OAs

It has been reported that feed additives with different functions and complementary mode of actions hold the most promising solution to replace antibiotics in animal feed mainly for three reasons: (i) all the beneficial effects of antibiotics are unlikely to be covered by an individual alternative; (ii) some alternatives possess a synergetic effect that may decrease the required dose considered as cost-effective; (iii) substituting the antibiotics must be an integrated approach that includes feeding, management and biosecurity rather than a supplementation of feed additives alone [172].

As for the synergy between feed additives, the combined use of hydrophobic EOs with lipophilic OAs has been considered the most promising method to substitute antibiotics for the potential synergistic and additive beneficial effects on the intestinal health and growth performance compared with individual EOs or OAs [181,182]. Some results of recent studies are illustrated in Table 7. The main mode of action linked to the synergic effects of OAs and EOs may be the modulation of the intestinal microbiota. However, the antimicrobial activities depend on the gram staining of bacteria as Gram-negative (G−) bacteria differ from Gram-positive (G+) bacteria with the respect to the structure of the cell wall. The cell walls of G+ bacteria are 90–95% composed of peptidoglycan allowing hydrophobic molecules (EOs) to easily penetrate the cells, acting on both the cell walls and the cytoplasm, causing a disruption of the structure and function of bacteria cell membranes [183]. This increase of the bacterial membrane permeability could facilitate the influx of OAs into the cytoplasm due the lipophilic nature of their undissociated form, disturbing the proton and associated anion concentrations in the cytoplasm [182]. As for phenolic compounds, once inside the cell, they can interfere with enzymes involved in the production of energy at lower concentrations and denature proteins at higher concentration. However, G− bacteria possess a different composition as their peptidoglycan layer is only 2–3 mm thick and composes only 20% of the dry weight of the cell. An outer membrane comprised of a double layer of phospholipids firmly linked by Braun’s lipoprotein to the inner membrane, lies outside of the peptidoglycan layer, making G− bacteria less permeable by providing an extra layer of protection and thus more resistant to EOs than the G+ bacteria [183]. Moreover, the “quid” provided by the core polysaccharides and the O-side chain allows these bacteria to be more resistant to EOs and other natural extracts possessing anti-microbial activities. Although the antimicrobial properties of long chain fatty acids (LCFA) are related to their potential to incorporate themselves into target membranes of G+ bacteria, promoting leakage of cellular protons and ions due to their lipophilic nature, the lipopolysaccharide (LPS) layer in the cell wall of G− bacteria prevents medium chain fatty acids (MCFA) and LCFA from crossing the cell membrane. Moreover, G− bacteria are also able to assimilate MCFA and LCFA into the cell and subsequently metabolize them per the β-oxidation cycle (i.e., *Escherichia coli*) and utilize short chain fatty acids (SCFA) as energy sources (i.e., *Salmonella*, *Escherichia coli*). These differences in the cell membrane compositions make EOs more powerful in the control of G+ bacteria compared to G− ones. However, OAs are reported to be more effective against G− bacteria than EOs [184] as small hydrophilic solutes of OAs are able to pass through the membrane via porin proteins but not the hydrophobic polyphenol molecules [172].

The mechanism of inhibition to microorganisms by OAs may be affected by several factors, including the reduction in pH, the ratio of the un-disassociated form of the acid, chain length, degree of branching and cell physiology/metabolism [189]. Indeed, the lipophilic nature of weak organic acid allows them to easily penetrate the plasma membrane and thus acidify the cell’s interior eventually killing the bacterium [172].

## 6. Conclusions

This review tried to gather the most recent available scientific information regarding the use of phytogenics in poultry nutrition along with their beneficial effects on performance, digestibility, microbiota, immune response, oxidant status, as well as egg and meat quality. Several studies have reported the promising effects of these feed additives when combined together or with organic acids; however, extra attention should be focused on the selection of active compounds to form potentially effective blends. Moreover, choosing the appropriate technique of protection as well as types and physiochemical properties of wall materials are the most critical aspects governing efficiency by controlling both the timing and location of the release of active compounds.

## Figures and Tables

**Table 6:** Effects of dietary PFA supplementation on immunity of broilers.

Feed Additive	Major Components	Dose, (mg/kg Diet)	Experimental Conditions	Immune Response	References
Curcumin EOs (PHY)	Curcumin (72%; CU) carvacrol (21.55 mg/g), thymol (18.76 mg/g) and cinnamaldehyde (27.62 mg/g) of PHY	50 100	Corn-soybean meal-based diet	↓ Total leukocyte and heterophils number in the CU and PHY + CU groups, ↓ lymphocytes in the CU group	[144]
EOs	Oregano oil (50 g), carvacrol (10 g), thyme oil (33.33 g), eucalyptus oil (50 g), thymol (5 g), eucalyptol(10 g), and acacia (Arabic gum) surfactant (27 g) in water up to 1 L	500	Challenge with virulent Newcastle disease virus+ vaccin against Newcastle disease (ND), the avian influenza (AI), infectious bronchitis (IB), and infectious bursal disease (IBD)	↓ Hemagglutination inhibition and viral shedding titres 1 wk after challenge ↑ ELISA antibody titre for IBD virus at the 28th d of age	[145]
Resveratrol	Polyphenols	300 and 600	Corn-soybean meal-based diet and chickens challenged with *Escherichia coli*	↑ Total Ig and IgG at d22 and total Ig and IgM at d 35	[84]
Cashew nut shell liquid and castor oil	Cardanol, cardol, and anacardic acid Ricinoleic acid	1500	Broilers challenged with *Eimeria* spp.	↑ Gene expression of TNF-α, IL-6 and IFN-γ and ↓ expression of IL-1 and COX-2	[146]
*Yucca schidigera*	Saponins	250	Corn-soybean meal-based diet Mixed Eimeria challenge	=Lymphocyte percentages to that of unchallenged birds on d7 p.i	[80]
EOs	Carvacrol (5%), cinnamaldehyde (3%), and capsicum oleoresin (2%)	100	Two control diets based on either wheat or maize	↓ CD40LG, IFN-γ and IL-6.	[86]
EOs	Cinnamon bark oil (CNO) Clove bud oil (CLO) Ajwain seed oil (AJO)	300 600 400	Corn-soybean meal-based diet. Broilers vaccinated against NDV at 5 and 18 d of age, and IBDV at 14 d of age.	↑ Antibody titres against NDV vaccine with CNO and CLO at 35 d of age	[147]
EOs	Carvacrol, thymol and cinnamic aldehyde	5000 and 10,000	Corn-soybean meal-based diet.	↑ Total erythrocyte counts, hemoglobin content and ↓ leucocyte count	[42]
Thyme powder	Major EOs (thymol (50.48%), γ-terpinene (11.03%), P-cymene (9.77%), and carvacrol (4.30%)), phenolic acids (salicylic acid (2450.03 ppm), ellagic acid (1240.42 ppm)) and flavonoid compounds	2000, 5000 and 8000	Corn-soybean meal-based diet.	↑ Lymphocytes, white blood cells, and IgG. ↓ TNF-α, IFN-γ, NF-κBP50 by all the doses. ↓ IL-6 by the dose of 8000	[87]
*Allium hookeri* (AH) roots Fermented root	Phenols	10,000 and 50,000 for both	Corn-soybean meal-based diet. LPS-induced young broiler chicken	↓ IL-1b with 1% AH root and 5% fermented root, TNFSF15 expression with fermented root (1% and 5%), and IL-8 with 1% fermented root supplementation	[148]
EOs	Oregano: (5%)	300	Corn-soybean meal-based diet.	↑ Secondary antibody titer and IgG titer, ↓ H/L ratio	[88]
*Artemisia annua*	Phenolics (44.24 mg GAE/g) and flavonoids (27.8 mg RE/g)	1000	Heat-stressed broilers	↑ Intestinal SIgA and IgG	[69]
Turmeric rhizome	Phenolic compounds (16.2 mg/g)	2000	Corn-soybean meal-based diet and broilers kept under chronic heat stress	↑ Total secondary antibody titer, and ↓ H/L ratio	[68]
EOs	Carvacrol (60.2%) and thymol (4%)	50 and 100 in water	Corn-soybean meal-based diet and broilers vaccinated with inactivated avian influenza and Newcastle disease (NDV)	↑ Antibody titer against NDV and avian influenza virus	[149]

SIgA: secretory immunoglobulin A; p.i.: post infection; ↓: increased; ↑: decreased: =: equal.

**Table 7:** Application of mixtures of phytogenics and organic acids with major physiological responses in poultry.

Mixture of OA+EO	Study Design	Main Findings	Reference
Citric (25%) and sorbic (16.7%) acids, thymol (1.7%), and vanillin (1.0%)	Type: male breeder chickens Dose: 500 g/metric ton diet Form: microencapsulated Duration: 15 days Conditions: without challenge	-Increased *Lactobacilliaceae*, *Clostridiaceae* and *Ruminococcaceae* abundance -Decreased Staphylococcaceae,	[185]
Thyme (4%), carvacrol (4%), hexanoic acid (0.5%), benzoic acid (3.5%) and butyric acid (0.5%)	Type: male Arbor Acres broiler chickens Dose: 500 mg/kg diet Form: Encapsulated Duration: 42 days Conditions: *Eimeria* spp. and *Clostridium perfringens*	-Improved FCR -Higher villus height and villus height/crypt depth ratio. -Reduced intestinal *C. perfringens* counts, liver *C. perfringens* carriage, and gut lesion scores. -Reduced serum fluorescein isothiocyanate dextran (FITC-D) concentrations. -Upregulated claudin-1, IGF-2 and A20 mRNA expression. -Downregulated TRAF-6, TNFSF15 and TOLLIP mRNA levels	[181]
Citric (25%, as fed) and sorbic acids (16.7%, as fed), thymol (1.7%, as fed) and vanillin (1%, as fed)	Type: Male Ross 308 broiler chickens Dose: 5 g/kg diet Form: Encapsulated Duration: 47 days Conditions: without challenge	-Improved growth performances -Improved gut morphology -Microbial control against *Clostridium perfringens*, *Enterobacteriaceae*, *Enterococci* and *Mesophilic* bacteria	[186]
Fumaric, sorbic, malic, and citric acids, thymol, vanillin, and eugenol	Type: Cobb 500 male broilers Dose: 300 g/t diet Form: Protected Duration: 42 days Conditions: *Eimeria* spp. and *Clostridium perfringens*	-Greater body weight gain -Higher apparent ileal nutrient and energy digestibility -Improved intestinal integrity with lower blood fluorescein isothiocyanate-dextran concentration -Improved intestinal macroscopic and histologic alterations -Greater expression of MUC2, CLDN1, and OCLN genes	[182]
Citric andsorbic acids, thymol, and vanillin	Type: By-product breeder chicks Dose: 500 g/metric ton diet Form: Microencapsulated Duration: 4 days Conditions: Without challenge	-Enhanced in vitro functional activity of peripheral blood leukocytes (degranulation, oxidative burst, and nitric oxide production)	[187]
Sorbic acid (200 g/kg), fumaric acid (200 g/kg), and thymol (100 g/kg)	Type: Roman laying hens Dose: 150 and 300 mg/kg diet Form: Encapsulated Duration: 21–30 weeks Conditions: Without challenge	-Increased laying rate with 150 mg/kg. -A linear increase in ileal villus height. -Increased mRNA relative expression of aminopeptidase, sodium-glucose cotransporter 1, and Na+-independent neutral amino acid transporter in duodenum and glucose transporter 2 in jejunum with 300 mg/kg. -Higher mRNA relative expression ofmucin-2 in ileum with 300 mg/kg. -Linear decrease of the secretory immunoglobulin in ileum A.	[188]

## Data Availability

Not applicable.
